# Women’s and Provider’s Moral Reasoning About the Permissibility of Coercion in Birth: A Descriptive Ethics Study

**DOI:** 10.1007/s10728-024-00480-4

**Published:** 2024-01-23

**Authors:** Johanna Eichinger, Andrea Büchler, Louisa Arnold, Michael Rost

**Affiliations:** 1 Institute for Biomedical Ethics, University of Basel, Bernoullistrasse 28, 4056 Basel, Switzerland; 2 Faculty of Law, Human Reproduction Reloaded|H2R, University of Zurich, Zürich, Switzerland; 3 Institute of Psychology, Friedrich-Schiller University Jena, Jena, Germany

**Keywords:** Childbirth, Obstetrics, Autonomy, Coercion, Medical ethics, Informed consent

## Abstract

Evidence shows that during birth women frequently experience unconsented care, coercion, and a loss of autonomy. For many countries, this contradicts both the law and medical ethics guidelines, which emphasize that competent and fully informed women’s autonomy must always be respected. To better understand this discordance, we empirically describe perinatal maternity care providers’ and women’s moral deliberation surrounding coercive measures during birth. Data were obtained from 1-on-1 interviews with providers (N = 15) and women (N = 14), and a survey of women (N = 118). Analyses focused on an in-depth exploration of responses to a question on the permissibility of coercion in birth whose wording was borrowed from a Swiss medical-ethical guideline. Reasons for and against a principle permissibility of coercive measures in birth were grouped into clusters of reasons to build a coherent explanatory framework. Factors considered morally relevant when deliberating on coercion included women’s decisional capacity, beneficence/non-maleficence, authority through knowledge on the part of providers, flaws of the medical system, or the imperative to protect the most vulnerable. Also, we identified various misconceptions, such as the conviction that a pathological birth can justify coercion or that fetal rights can justifiably infringe on women’s autonomy. Information and education on the issue of coercion in birth are urgently needed to enable women to fully exercise their reproductive autonomy, to prevent long-term adverse health outcomes of women and children, and to reconcile the medical vigilance which has lead to a reduction of perinatal morbidity and mortality with women’s enfranchisement in their own care.

## Introduction

Numerous studies have evidenced that limitations of autonomy are crucially linked to women’s negative birth experiences [3, 26, 31, 48]. One way that deprivation of autonomy actualizes is coercion, which hence qualifies as a form of mistreatment in birth [3]. While noting that different forms of coercion are not entirely mutually exclusive, the following distinctions can be made: (a) formal coercion—acting physically on a patient’s body (e.g. forcing into lithotomy position, strapping to bed), (b) informal coercion—acting more subtly on a patient’s mental state (e.g. intimidation, manipulation, withholding information), and (c) coercive environment—consciously limiting a patient’s array of infrastructure-related options (e.g. not affording an available bathtub, locking the door) [15, 31, 40]. For example, in a Canadian sample 1 in 10 women reported feeling coerced into accepting options recommended by providers [47]; in a US sample half of women who preferred vaginal birth over caesarean-section were not afforded this option [8]; in a Swiss sample more than 1 in 4 women experienced informal coercion [31]; in a Nigerian sample more than 1 in 6 women were restrained or tied down during labor [32].

Any form of coercion attempts to override a patient’s self-determination. Related to this, pathological birth situations were reported to lead to, if not to justify coercive measures which are seen as a means to gain compliance from women and to adhere to guidelines [21, 36, 37]. Coercion in birth is not only recognized as a violation of human rights [52], but is antithetical to quality care [50], and often causes adverse psychological outcomes [9, 31], which ultimately affect mother–child-bonding and child well-being [39], as well as parental couple relationships [33]. In contrast to its widespread occurrence, both ethical and medical associations (e.g. World Health Organization, American College of Obstetricians and Gynecologists, Nuffield Council on Bioethics, Swiss Academy of Medical Sciences) universally reject any coercive measures being imposed on women (with decision-making capacity) during birth and emphasize the right to autonomy as well as the deduced requirement of informed consent, thereby codifying the recognition of women’s reproductive rights [27, 40, 45, 51]. Also, these publications indicate that coercion has generally garnered attention in obstetrics.

From a swiss legal perspective, reproductive autonomy and bodily integrity are affected whenever a physician orders a pregnant woman to undergo a particular treatment to protect the embryo. According to the Swiss Constitution, reproductive autonomy is a fundamental right and part of the constitutionally protected right to personal freedom and bodily integrity (Art. 10) [13]. It follows, that for restrictions to be justified they need to pursue a public interest or protect the fundamental rights of a third party, and they need to be proportionate. However, there are uncertainties regarding the scope of reproductive autonomy which are essentially attributable to the fact that the appropriate legal treatment of the embryo has not been fully determined. Technical developments have exacerbated these uncertainties. There is now increasing scope for carrying out medical interventions on the embryo itself. This has shifted perceptions significantly. The foetus is increasingly seen as an entity separate from the mother, and a patient in his or her own right. The public discourse refers to the “anticipated well-being of the child” and to the “unborn child” as a “patient” to justify infringements on a woman’s reproductive autonomy and her corporal integrity. Nevertheless, legal norms in Switzerland and many other countries hold that legal personality does not begin until birth. The unborn have no fundamental rights. That also means that a woman’s right to reproductive autonomy and bodily integrity is not matched by similarly robust rights on the part of the foetus.

Any prenatal medical intervention is performed on the body of the woman giving birth. Birth is a significant moment in the status of a human life. In many jurisdictions, it marks the beginning of legal personhood. Of course, a woman’s autonomy does also encompass the choice of delivery method used and she can decline medical assistance in the birth process. She is the patient and any action taken requires her consent, which she is entitled to withhold, even if the suggested action can be deemed to serve a useful purpose and medical indications support it. The prerequisites for a valid consent are a woman’s decision-making capacity and full information about benefits and risks of the intervention and possible alternatives to it. The woman may have a moral duty to tolerate a bodily intervention which benefits the health of the future child, she does not, however, have a legal obligation to do so.

Nevertheless, open legal questions remain regarding the extent to which a pregnant woman can be required to submit to certain obstetric procedures or indeed to give birth by caesarean section. For example, it can become necessary to change the method of delivery. This may be the case if there is deterioration in the fetal heart sounds or if persistent lack of oxygen would result in damage to the fetal brain, so that a caesarean section needs to be considered. There is some legal uncertainty surrounding the birth-giving process, for within the realm of Swiss Criminal Law (Art. 116) [44]—different from the Civil Law (Art. 31) [43]—personhood begins at the onset of labour. Yet, the cardinal principle that no medical interventions are permitted in cases of a person capable of judgment does not consent to them holds true also during the process of giving birth. Coercive delivery by Caesarean section is tantamount to injury to the bodily integrity of the woman concerned. If the law does not place any obligation on women to subject themselves to bodily interventions benefiting the child neither during their pregnancy nor after birth, to deprive her of the right to bodily integrity during the hours surrounding a birth not only creates an incoherent value hierarchy, it also impinges on the woman’s dignity at a time when she is especially vulnerable. Finally, whether medical intervention occurs in the first place, and what form it takes if it does, varies considerably—not only according to the cultural and social setting but also based on personal attitudes to risks (eg. life, health). As birth has become increasingly medicalised, women are finding it ever more difficult to oppose the use of technology. To then transpose their decisions into the context of criminal law is hardly compatible with the principles of reproductive autonomy.

In light of the frequently observed divergence between obstetric practice and ethico-legal standards it is of paramount importance to understand the reasoning of involved parties (e.g. providers, women) underlying the view that coercion in birth is (im)permissible. It is imperative to first analyze their normative reasoning descriptively to identify their reasons for and against coercive measures. Only in a second step, the particularities of their reasoning can be addressed normatively. Deliberately departing from a normatively neutral stance, descriptive ethics describes the manifold aspects and manifestations of morality as a natural phenomenon (e.g. people’s moral behavior, values, principles) [12]. Descriptive and normative ethics are inextricably linked and mutually constitutive in their attempt to answer the question “what is morality?” [12]. Along similar lines, Hämäläinen argues that “philosophical ethics cannot be pursued in meaningful ways without substantial descriptive work” and that “the main reason why the projects of descriptive ethics are left to others [e.g. social scientists] is that there is in today’s philosophical ethics too little appreciation of the philosophical import of descriptive work and the philosophical hazards involved in such work”[25, p. 2]. Sharing this conviction, we applied a descriptive ethics lens to providers’ and women’s moral reasoning concerning the permissibility of coercion in birth. In doing so, our analysis aimed to serve the descriptive task of ethics, namely to provide “rich and accurate pictures of the moral conditions, values, virtues, and norms, under which people live” and which drive their behavior [25, p. 1]. The reasons for and against coercion in birth identified by our analysis can serve as points of leverage to dismantle coercion by addressing possible fallacious arguments (e.g. formal logic) or premises that do not match current legal or ethical standards (e.g. premise on the moral status of the fetus) or that are not empirically true (e.g. coercion does no harm to the child). Moreover, they can advance the ethical discussion surrounding coercion in obstetrics by providing a comprehensive list of factors considered morally relevant for this subject. Most importantly, however, our analysis ultimately contributes to improving lived birth experiences.

## Methods

### Study Design

Analyzed data were obtained from two different studies, which were part of a larger mixed-methods project addressing decision-making in birth in Switzerland: (1) 1-on-1 interviews with providers and women, (2) an online survey of women. The present analysis focused on an in-depth exploration of providers’ and women’s responses to the following question on the permissibility of coercion in birth that was included both in the interview-guide and in the survey: “Do you think it can be, under some circumstances, permissible to impose—during birth—a medical measure on a pregnant woman with capacity who can recognize and assess the consequences of her actions and consciously accepts adverse effects for herself and her child?”. The exact wording was taken from the Swiss Academy of Medical Sciences’ (SAMS) medical-ethical guideline “Coercive measures in medicine” [6, p. 19].

Study documents were reviewed by the responsible ethics committee (Ethikkommission Nordwest*-* und Zentralschweiz; EKNZ). The EKNZ stated that the projects do not fall under the remit of the Swiss Human Research Act (Art. 2) because, for the survey, data were collected anonymously, and, for the interviews, no personal (i.e. health-related) data were collected and data collection was anonymous. Also, interviewing health professionals does not require ethical approval in Switzerland. Hence, ethical approval was not needed. Still, the EKNZ issued a declaration of no objection (Req-2019-00017) and stated that the project fulfills the ethical and scientific standards for research with humans (Art. 51, Swiss Human Research Act).

### Participants

In total, we analyzed interview responses from 15 providers and 14 women and survey responses from 118 women, resulting in a total sample of N = 147 (tab.1). We interviewed women either before or after birth (before: during pregnancy; after: within 12 months postpartum) and surveyed women twice, before and after birth (before: last trimester; after: 6–16 weeks after expected date of delivery). However, 39 women did not fill in the post-birth survey. Informed consent was obtained prior to interviews and surveys.

**Table 1:** Participants’ characteristics (N = 147)

Interviews	
Providers (n = 15: n = 8 midwives, n = 5 physicians, n = 2 doulas)	
Age	M = 41.5 (SD = 9.7), Min = 27, Max = 54
Gender (woman)	93.3%
Work experience (years)	M = 14.5 (SD = 9.6), Min = 1, Max = 34
Women (n = 14: n = 3 pre-birth, n = 11 post-birth)	
Age	M = 35.0 (SD = 4.0), Min = 29, Max = 43
Place of birth^1^	71.4% hospital, 14.3% birth center, 14.3% home
Nr. of previous pregnancies	50% zero, 50% one
Survey^2^	
Women (n = 118: n = 79 pre- and post-birth, n = 39 pre-birth)	
Age	M = 32.0 (SD = 3.95), Min = 21, Max = 45
Intended place of birth	50% hospital, 41% birth center, 7% home, 2% other
Nr. of previous pregnancies	51% zero, 31% one, 13% two, 5% more than two

^1^Actual place of birth for women interviewed after birth and planned place of birth for women interviewed before birth

^2^As stated in pre-birth survey

### Recruitment

For the interviews, we recruited providers and women from birth hospitals and birth centers; recruitment is described elsewhere [36]. For the survey, recruitment of women was the same as for the interviews. Additionally, the link of the online survey was shared through newsletters of the Swiss Federation of Midwives and of the Swiss Society for Gynaecology and Obstetrics.Data collection was conducted between 06/2020 and 01/2021.

### Study-Tools

A semi-structured interview-guide was employed to explore providers’ attitudes towards decision-making in birth. It consisted of 13 main questions, capturing the following areas of interest: intra-team collaboration, ethical principles associated with intrapartum care, decision-making, informed consent, autonomy, decisional capacity, guidelines, and coercion. A second semi-structured interview-guide was employed to explore women’s attitudes towards decision-making in birth. It consisted of 13 main questions, assessing the following areas of interest: antenatal preparation for birth, preferred place of birth, birth experience, changed attitudes due to previous birth experience(s), ethical principles associated with intrapartum care, decision-making preferences, coercion. The survey was comprised of the following main parts: demographics, attitudes towards and preferences for birth and decision-making in birth, personality-related constructs, and the birth experience. Further details on recruitment and employed study tools have been reported elsewhere [36, 37]. The present analysis exclusively addresses the above-mentioned question on the permissibility of coercion.

### Data Analysis

**Fig. 1:**
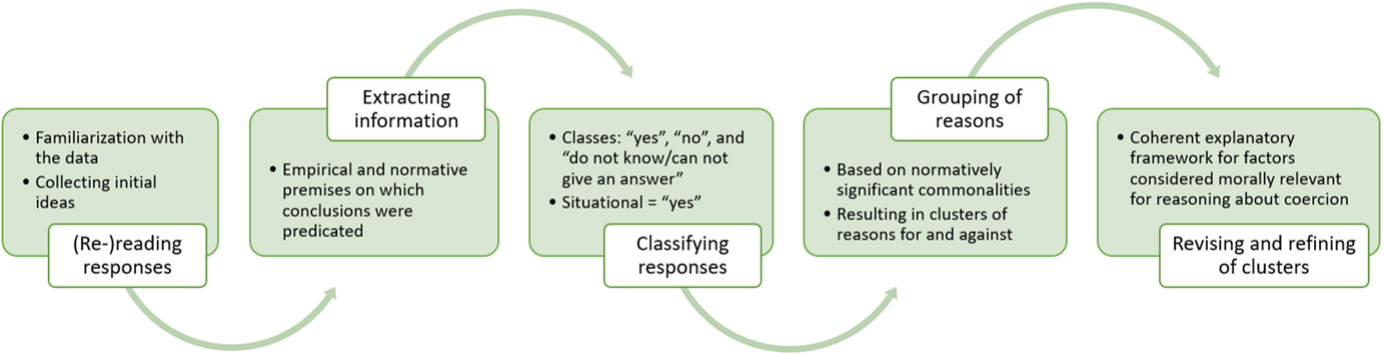
Descriptive-analytic process

In an effort to better understand the sometimes observed discordance between normative imperatives (i.e. legal documents, medical-ethical guidelines) and actually unfolding obstetric practice (i.e. application of coercive measures), the present study empirically describes the morality (e.g. values, principles, premises, norms) surrounding coercive measures in birth brought forward by providers and women. Analysis followed a multi-stage process (Fig. 1) to build a coherent framework of factors considered morally relevant for reasoning about the permissibility of coercion in birth. It has to be noted that responses which included a reference to some sort of dependency of the permissibility of coercion (e.g. “depends on the situation”, “depends on the risk”) were classified as “yes”, since such responses indicate a principle approval of coercion (Fig. 1).

## Results

### Principal (Dis)approval Rates

**Fig. 2:**
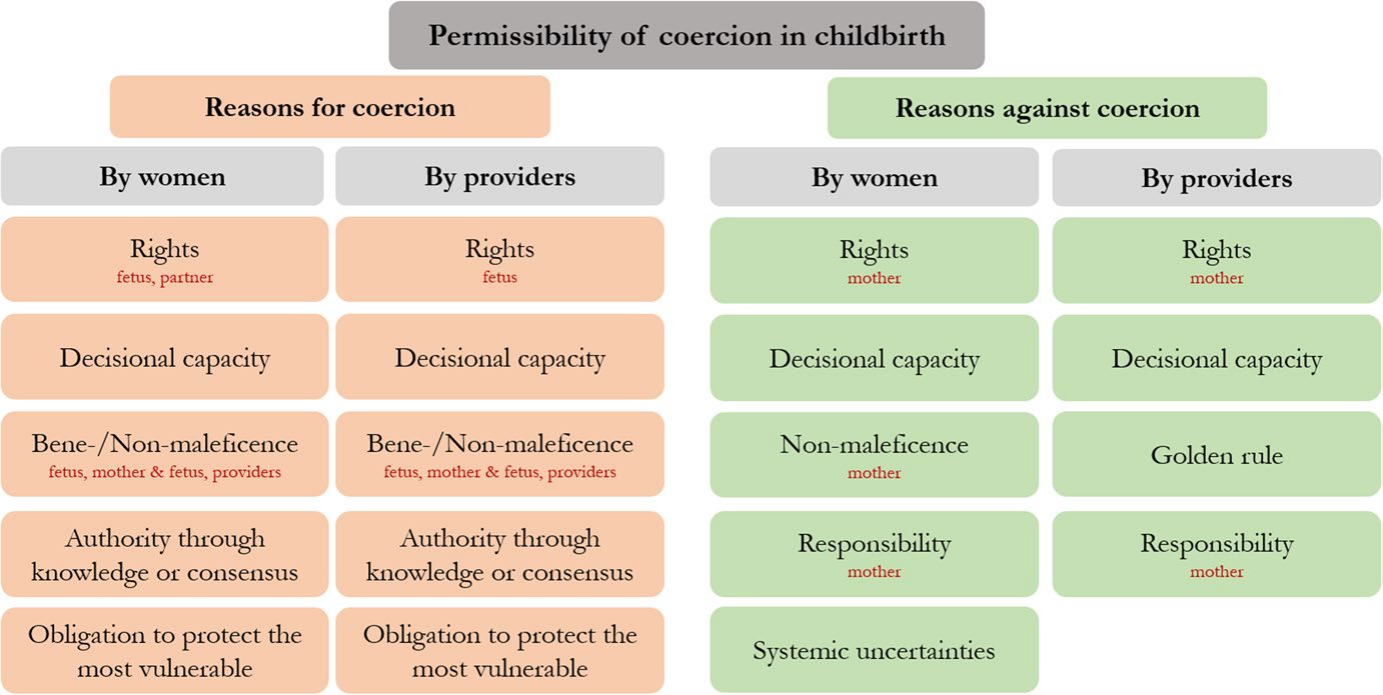
Factors considered morally relevant for reasoning about coercion

Overall, a relative majority of women and providers approved coercion in birth under some circumstances. More precisely, 48.5% (64/132) of women thought that it is permissible to impose medical measures on a birthing woman with capacity to recognize and assess the consequences of her actions and consciously accepts adverse effects for herself and her child; 37.9% (50/132) thought it is not permissible, and 13.6% (18/132) did not know. For providers, 46.7% (7/15) thought it is permissible, 40.0% (6/15) thought it is not permissible, and 13.3% (2/15) did not know. We identified clusters of reasons for and against coercion expressed by women and providers (Fig. 2, Tables 2, 3).

**Table 2:** Classification of reasons for coercion

Clusters	Specific instances	Quotes
Reasons for coercion—Women		
Rights	Fetus: right to life and health	*But in principle, I think the baby has a right to be protected if the negative consequences are very severe*
	Partner: right to have a say	*I also think the father needs to have a say when it comes to the child*
Decisional capacity	Limited or questionable	*Since (…) the mother is in exceptional circumstances. She might decide differently if she were not in labour* *A woman giving birth may not be fully capable of judgement* *I think we women judge differently during birth, labour, pain than without or afterwards*
Beneficence and Non-maleficence	Fetus: life and health	*Because (…) the safety and outcome for the newborn is the top priority!!!!* *If the child is threatened with harm, this must be avoided. The child cannot stand up for itself*
	Mother and fetus: life and health	*When it comes to health or a life-threatening situation, whether it's the baby or the mother, I think the doctors have to act* *The best for mother and child*
	Priority of somatic medical outcomes	*Because doctors are principally obliged to save lives*
	Providers	*Medical staff also have to be able to "live" with the situation without blaming themselves for not having done everything for mother and child*
Decisional authority through knowledge or consensus	Knowledge: medical expertise, medical experience	*No birthing woman can assess all the factors as good as the professional observing from the outside!* *Medically, many cannot assess the implications. Especially in a stressful situation* *I think the midwife or doctor is more experienced and only wants the best for mother and child. With today's information from the internet, many feel they have real knowledge. However, the information is often not correct*
	Consensus between staff and accompanying person	*If the accompanying person is also involved in the decision-making process possibly yes*
Protecting the most vulnerable	Advocate of child	*If the child is threatened with harm, this must be avoided. The child cannot stand up for itself* *The child could not choose for itself, so the medical staff should advocate for the child*
Reasons for coercion—Providers		
Rights	Fetus: human rights	*A child, for example, is a human being as soon as the birthing situation begins and not only when it is born. And that means that human rights also begin with the birthing situation, although the child is not even there yet… if one has the feeling that we have to do this now, because otherwise something bad will happen and the woman does not agree, then we are allowed to still act in the interest of the child* *I find birth difficult, because the major point for me is a little bit of…, yes, it goes a bit in the direction of child protection. It is not only one patient*
Decisional capacity	Limited or questionable	*And with birth it is just, I also think that, pain and also fear, the woman is not even in her normal emotional state, so this might influence her fear and pain. And regarding certain decisions I just ask myself "what about the child?", can she really assess that?*
Beneficence and Non-maleficence	Mother and fetus: life and health	*If the child is really at risk. That it would die in the womb at this point* *But of course it is certainly usually better that you then perhaps make a decision, for the sake of the child, for example*
	Priority of somatic medical outcomes	*The job is to keep the woman and child alive*
	Providers	*The problem is that I myself can't stand to watch and let it happen… Yes, we have to endure it and there's no one there to endure me. So I put it away somewhere so that the outside doesn't notice and inside you're sometimes just happy if you're no longer reminded of it* *I think, yes, when it is really a matter of life and death or when it is a matter of the child, who is incapable, that is sometimes indicated, because we are also involved, we also have to live with it when we let this woman die and bleed to death. That is also a huge trauma for us. … there are situations where I would do that, because I couldn't live with that trauma either. Bad luck for the woman—I don't know*
Decisional authority through knowledge and consensus	Knowledge: medical expertise	*But if, (…) birth happens very quickly, she is very surprised, she presses her legs together, doesn't let the child come out because she is simply taken by surprise by the situation and there are also… Yes, you can forcibly hold her legs so that the child comes out (…) And in these situations, I think it should be possible, because the staff is clearer-headed at this moment and also knows more that it simply can't be done now, the child simply has to come out now, no matter what kind of crisis situation this woman is in now*
	Consensus between staff and accompanying person	*If I have a partner there who tells her "look, our baby will die otherwise", then you can also coerce her, yes*
Protecting the most vulnerable	Advocate of child	*Yes, but the woman can express herself, can talk, and the baby cannot talk yet. And that's why the obstetric staff has to talk for the child from time to time*

**Table 3:** Classification of reasons against coercion

Reasons against coercion—Women		
Rights	Mother: dignity, human rights, parental right to be surrogate DM	*She has a right to self-determination, for herself and her child* *Parents are responsible for themselves and their child. Even anti-vaccinationists are not forced to vaccinate their children. And Jehovah's Witnesses are allowed to forbid blood transfusions on their children, even though lives can be saved by doing so [mistaken belief]. For me, the above question belongs in the same category* *This is not possible under any circumstances. It violates human rights. As long as she is competent, the woman has the right to decide and should not have to fight for it*
	Bodily integrity	*It is her body. It does not suddenly become the property of a doctor/midwife *etc*. when she enters hospital*
Decisional capacity	Capable of judgement	*Because the mother is just as capable of judgement during birth as she is at any other time* *Yes, if she understands and if she is clear-minded and says: No, I don't want that. Then that should basically be accepted, I think*
	Has been fully informed	*I assume that she was informed about the consequences and thus consciously says yes to them. Then this should be respected* *After the expectant mother has been well informed, her decision has to be respected* *The woman decides. It is always a risk assessment, certain negative consequences are rare and even rarer are mothers who "accept" them. A well-informed and carefully cared for woman will also make the right decision for her and her child. If not, something is wrong and one should ask oneself whether the underlying circumstances might not be good*
	Embodied knowledge	*A woman in labour feels what is right* *Because medical wisdom is not always better than maternal intuition* *Well, because then the woman has to decide, you have to explain to the woman that it is really dangerous now and then the woman will most likely make the right decision for herself and for her child. Because it is her child. And the mother knows more than the others and of course*
Non-maleficence	Mother: Harmful Consequences of coercion	*Negative consequences due to coercion* *Coercion can have traumatic consequences* *Paternalism is violence and can cause long-term trauma*
Responsibility	Mother has to live with consequences	*But then she must also bear the consequences* *Because the woman has to live with this decision all her life*
	Mother is responsible	*The mother takes responsibility for herself and for her child*
Systemic flaws	Medical risk assessments possibly wrong	*Doctors don't know everything either, [it is] often a weighing of risks*
	Interventionist culture in hospitals	*I think today caesarean sections are performed too quickly* *There are far too many interventions* *I think basically the doctors are also less empathetic first of all, and just look for a quick solution*
Reasons against coercion—Providers		
Rights	Bodily integrity	*The woman should have the last word. It is her body and she must be able to decide for herself*
	Law: bodily harm	*Yes, but I think the episiotomies in the past… well, we often discussed in midwifery training that this is active intentional bodily harm, which would be punishable in any other setting*
Decisional capacity	Has been fully informed	*Yes, if she refuses the procedure despite being informed, then that would have to be accepted* *And then we see them regularly, document each time that we have informed them with our legal department*
Golden rule	Reciprocity	*I personally don't want anything to happen to me that I don't want. (…) That's also how I consider my patients: (…) "well, the same applies to them"*
Responsibility	Mother has to live with consequences	*Yes, if the woman wants to bear these consequences, then it is justified [not to force her]*
	Mother is responsible	*Actually, the woman has to take the responsibility*

It has to be noted that 24 out of the 82 women (29.3%) with pre- and post-birth responses provided inconsistent responses (i.e. post-birth response did not match pre-birth response). Given the three possible classes of responses (i.e. “yes”, “no”, “don’t know”), six inconsistent response patterns were possible and occurred as follows: “don’t know/yes” two times, “don’t know/no” three times, “yes/don’t know” seven times, “no/don’t know” two times, “yes/no” three times, and “no/yes” seven times; resulting in six women who changed their opinion to “no” (25.0%), nine to “yes” (37.5%), and nine to “don’t know” (37.5%) after having given birth. Inconsistent responses that contained one “yes” (either pre- or post-birth) were classified as “yes” (n = 19); responses with “no” and “don’t know” were classified as “no” (n = 5).

### Reasons for Coercion in Birth

Women’s and providers’ reasoning in favor of coercion was similar and can be grouped as follows (Table 2). Both women and providers referred to various rights. While providers exclusively talked about rights of the fetus, women also acknowledged the partner’s right to have a say, ultimately justifying coercion. Furthermore, although the posed question explicitly described a woman with capacity of judgement, both women and providers argued that women’s decisional capacity might still be limited and therefore coercion is permissible. Additionally, both women and providers referred to the principles of beneficence and non-maleficence. While both participant groups mentioned life and health of the fetus or of the mother and the fetus as well as traumatic consequences for providers, women also mentioned that it is in a woman’s own best interest to have a healthy child. Women and providers also advocated for prioritizing somatic medical outcomes over women’s autonomy. Moreover, both women and providers ascribed decisional authority to providers which justifies coercion and can stem from two sources: medical expertise or experience on the part of providers, and a consensus between providers and the accompanying person. Lastly, both women and providers argued that providers have to protect the most vulnerable (i.e. fetus) and thus coercive measures are permissible.

### Reasons Against Coercion in Birth

Women’s and providers’ reasoning against coercion in birth was less congruent as compared to the reasoning brought forward by the group arguing in favor of coercion. First, both women and providers referred to the woman’s rights (e.g. bodily integrity, human rights, right to be surrogate decision-maker). Second, both women and providers emphasized that coercion is impermissible when a woman is capable of judgement. Third, only women referred to non-maleficence as a guiding principle, stating that coercion can have harmful consequences for women. One provider demanded the golden rule to be applied (i.e. treat others the way you want to be treated). Fourth, both women and providers referred to the maternal responsibility which not only means to be responsible, but also to bear the consequences. Lastly, women stressed that even medical professionals’ risk-assessments can be mistaken and that hospitals suffer from an interventionist culture and, hence, coercive measures should not be applied.

## Discussion

Mapping factors considered morally relevant for coercion in birth is a prerequisite for a meticulous normative analysis. For example, considered moral judgements and moral intuitions of relevant agents can be fed into a process of reflective equilibrium [46], which was originally developed as a method of doing moral philosophy [34], but which has also been widely applied as a discussion and decision model facilitating case-based reasoning and justifying decisions regarding concrete ethical issues [35]. More recent versions of reflective equilibrium consist in working back and forth among the following four relevant groups of moral beliefs: considered moral judgements of relevant agents, morally relevant facts, ethical principles, and both descriptive and normative background theories [46]. By equilibrating this quadratic set of moral beliefs, the moral justification is not founded in “secure, incorrigible foundations outside of our processes of reflection, but rather in the coherence of all flotsam and jetsam of our moral life” [29, p. 47]. As such, our descriptive ethics analysis can help anchoring future normative analyses of coercion in birth in existing moral beliefs of persons other than the ethicist(s). Notably, the fact that in our study a relative majority of women and providers approved coercion in birth under some circumstances contrasts with existing legislation and relevant medical ethical guidelines [5, 27, 40, 45]. However, it fits into the global picture that numerous empirical studies from various countries have highlighted that many women experience violations of autonomy during birth [4, 30, 38, 48].

Many women and providers justified coercion in birth by referring to the concept of decision-making capacity. Two misconceptions are evident in this context. First, although the question used in the interviews and survey explicitly assumed women with capacity, the latter was frequently questioned, even by women themselves. The mere reason provided for the assumption of limited decision-making capacity were the exceptional physical and emotional states birthing women are often in, rather than the ascription of incapacity based on a rigorous evaluation of the abilities underlying decision-making capacity (i.e. cognitive, evaluative, decisional, expressive) [41]. The standards for decision-making capacity during birth seem to be set differently and higher than in other areas of medicine, which, however, lacks any legal and ethical basis and can be seen as a form of paternalism and oppression of (birthing) women [36]. Moreover, precisely these exceptional states, which are cited as a reason for questioning birthing women’s decision-making capacity, are conducive to an effective physiologic birthing process [7, 11]. The second misconception which emerges in our interviews related to decision-making capacity is that often as soon as a birthing situation becomes pathological, coercion was said to be justified. However, overriding autonomy can only be justified if a birthing woman lacks decision-making capacity and not simply because she does not consent to a measure deemed necessary by providers [5, 41]. Apparently irrational refusal of recommended care options in health-threatening situations does not eo ipso equate to a lack of decision-making capacity [42].

In defense of coercion, it is often argued that providers hold the (more) objective knowledge. On the one hand, there is of course a medical knowledge asymmetry between women and providers (as generally between patients and providers). On the other hand, this should not result in a power imbalance between women and providers which would represent a form of epistremic injustice [14]. Also, the idea of providers’ assessments being always objective and correct conveyed by such justifications is contestable. Introducing the concept of “authoritative knowledge”, the anthropologist Brigitte Jordan analyzed how in birth a structural superiority of the medical system prevails and other systems of knowledge are disregarded [22, 23]. Furthermore, “maternity care providers are bound to the limits of a medicalised model of care, and socialised into the risk-focused approach of this model” [41, p. 338, 42]. Therefore, the situation described in the question used is often averted proactively, either by tailoring “information to ensure the selection of what the health care expert considers the best choice” [43, p. 267], or by evoking fear through manipulating and intimidating statements such as “if you want your child to die …” [31]. In this way, coercion actualizes beforehand at an informal level. The birthing woman consents and physical coercion has been avoided.

Related to this, many of the justifications made in support of the use of coercion also reveal a strong focus on the somatic dimension of health. Whenever “best interest”, “outcome” or “health” was mentioned, participants referred to (short-term) somatic health. Possible other health-related outcomes, such as the mental health of the mother, mother–child-bonding, parental couple relationships, or future reproductive choices are neglected and appear to be mostly unknown. Paradoxically, possible negative psychological consequences for providers are presented as an additional justification for coercion. Thus, overall, providers seem to be perceived as more accountable in regard to the protection of health and life than in regard to the use of coercive measures. It seems worse not to have protected health of the entrusted than not to have protected maternal autonomy, so that in (perceived) risk situations the principle of non-maleficence is given more weight than the principle of autonomy [36, 37]. Yet, providers are dually accountable, that is they have to respect autonomy and protect health and, in case of an unsolvable conflict between autonomy and non-maleficence, they have to prioritize autonomy [5, 27, 40, 45].

In face of the challenge of decision-making in birth, the legal scholar Abrams argues that a decision-making framework is applied which elects the outcome that minimizes any, even minor, fetal risk [1]. The author points out that such a fetal-focused framework perpetuates an illusion of autonomy in birth and concludes that “law [and ethics] standards should explicitly govern not just the ‘what’ of childbirth outcomes, but the ‘how’ of childbirth decision-making (…) to ensure that women ‘s autonomy is actual and not illusory” [1]. Correspondingly, it has been argued that framing moral problems of birth as maternal–fetal-conflict is a misguiding conception which disregards women, results in a baby-centric bias, and commonly turns providers into allies of the fetus [49]. This is also apparent in the results of our study in that mothers are never mentioned on their own under the aspect of *beneficence*, but only—if at all—in combination with the fetus. Such a conflict-lens abets a contest between women’s autonomy and fetal beneficence, which, in turn, may contribute to strained decision-making, as argued by the bioethicist De Vries [49]. Hence, the author proposes to replace autonomy with respect, which cannot be ignored in the name of beneficence or non-maleficence and which creates an ethical obligation to honor women’s preferences, fears, and uncertainties [49]. Both bioethics and law have to self-critically assess their contributions to coercion in birth (e.g. by putting fetus and woman in opposition, deprioritizing respect, avoiding the subject all together) in birth.

Many respondents voiced that they found the question difficult to answer or answered “don't know”. This suggests that providers facing such difficult situations in obstetric practice may suffer from moral distress [20]. In fact, available research indicates that one growing challenge for midwives in Switzerland is moral distress, amongst others due to institutional limitations of women’s autonomy and quality of care [28, 29]. Although the ethico-legal background is unambiguous, many of the interviewees seem to work in uncertainty or ignorance about the legal and ethical standards underlying their work. Both moral distress and uncertainty may be exacerbated by the absence or the marginal role the issue of coercion in birth plays in several medical-ethical guidelines on coercion in medicine [15, 40]. This is surprising given the huge number of people affected by this issue (e.g. woman, companions, partners, children) and the growing body of evidence on mistreatment (e.g. coercion) in birth [4, 30, 38, 48]. Here, the question has to be raised how existing patriarchal norms and power structures may contribute to the invisibility and marginalization of women’s experiences of coercion.

Considering practical implications of our study, we first advocate that it is of immense importance that providers are well aware of the ethical and legal bases, for example that the topic of coercion is (more extensively) dealt with in education and training. Furthermore, also women and their partners should know their rights better. This could be addressed through a systematic implementation of this subject in birth preparation courses in hospitals or birth centers. Should autonomy violations nevertheless have occurred, it would be helpful that low-threshold possibilities and sensitive mechanisms exist for the women concerned to denounce them. It is known that civil justice systems are mostly of limited value in addressing mistreatment in birth (e.g. coercion) and, hence, render redress out of women’s reach [10]. Against this backdrop, the concepts of obstetric violence and mistreatment have been welcomed as a first step and an epistemic intervention, that is “by rejecting the normalization of reproductive oppression, [it] constitutes a refusal of epistemic frames that silence, diminish, erase, and devalue alternative and embodied forms of reproductive knowledge and agency” [50, p. 104, 51]. However, existing law standards have to catch up to these new realities.

## Limitations

Self-selection-bias resulting in interviews and survey with participants who hold strong attitudes towards the topic. In fact, women with a preference for birth centers were overrepresented in our sample. Moreover, we only interviewed and surveyed participants from the German-speaking part of Switzerland. Participants from other major language regions might have reasoned differently. Lastly, the wording of our analyzed question on coercion applied a conflict-lens which might have obscured the interconnection between the woman and the fetus and, thus, biased responses. However, we used the exact same wording as in the medical-ethical guideline of the Swiss Academy of Medical Sciences, since the latter is legally binding for Swiss providers.

## Conclusion

Our study has mapped various factors considered morally relevant by providers and women when deliberating on the permissibility of coercion in birth, including women’s decisional capacity, beneficence and non-maleficence, authority through knowledge on the part of providers, flaws of the medical system, or the imperative to protect the most vulnerable. Also, we identified various misconceptions, such as the conviction that a pathological birth can justify imposing coercive measures on a woman with capacity or that fetal rights can justifiably infringe on women’s autonomy. This apparent discrepancy between several statements and existing medical-ethical guidelines and legislation urgently calls for information and education on the issue of coercion in birth to enable women to fully exercise their reproductive autonomy, to prevent long-term adverse health outcomes of women and children, and to make “women’s enfranchisement in their own care rest easily with the medical vigilance which has helped to reduce perinatal and maternal morbidity and mortality” [52, p. 1144].

## Data Availability

Since participants have not provided consent to share their data, study materials cannot be made openly available. However, we will share portions of the study material relevant for the manuscript upon reasonable request. Please contact the corresponding author.
